# Surveillance and Genomic Characterization of Influenza A and D Viruses in Swine, Belgium and the Netherlands, 2019–2021

**DOI:** 10.3201/eid2907.221499

**Published:** 2023-07

**Authors:** Anna Parys, Nick Vereecke, Elien Vandoorn, Sebastiaan Theuns, Kristien Van Reeth

**Affiliations:** Ghent University, Merelbeke, Belgium (A. Parys, E. Vandoorn, K. Van Reeth);; Ghent University and PathoSense BV, Lier, Belgium (N. Vereecke, S. Theuns)

**Keywords:** influenza, influenza A virus, influenza D virus, swine, genetic characterization, subtyping, viruses, Belgium, the Netherlands, respiratory infections, zoonoses

## Abstract

During 2019–2021, we isolated 62 swine influenza A viruses in Belgium and the Netherlands. We also detected influenza D in pigs in the Netherlands. The ever-changing diversity of influenza viruses and the identification of influenza D emphasize the need for more virus surveillance.

Three influenza A virus (IAV) subtypes circulate globally in swine: H1N1, H1N2 and H3N2. Within each subtype, multiple hemagglutinin (HA) and neuraminidase (NA) lineages or clades cocirculate. In Europe, there are 4 swine IAV HA lineages: the HA-1A classical swine lineage including influenza A(H1N1)pdm09 virus (pH1N1), HA-1B human seasonal lineage (H1hu), HA-1C Eurasian avian lineage (H1av), and European human-like H3 lineage (*1*). In addition, there are 4 NA lineages: pH1N1, N1av, A/swine/Scotland/410440/1994-like (N2s), and A/swine/Gent/1/1984-like (N2g). Swine IAV lineages have continued to evolve through exchange of genome segments (reassortment) and mutations in the viral surface proteins (drift); these processes resulted in multiple genotypes with gene segments of swine, avian, and human origin. 

A new genus, influenza D virus (IDV), was identified in pigs in Oklahoma in 2011 (*2*). Two IDV lineages, D/swine/Oklahoma/1334/2011 and D/bovine/Oklahoma/660/2013 (D/660), have been identified in swine and cattle in Europe on the basis of the hemagglutinin-esterase fusion protein (HEF). In this study, we identified the prevailing swine IAVs circulating in Belgium and the Netherlands during 2019–2021 and determined their genotypes by whole-genome characterization. Furthermore, we described the isolation and characterization of an emerging swine IDV in the Netherlands.

## The Study

We started with 152 samples that had tested IAV-positive by reverse transcription PCR by Animal Healthcare Flanders (Torhout, Belgium) or through random viral and bacterial metagenomics analysis by PathoSense (Lier, Belgium) (*3*). The samples were submitted to the diagnostic laboratories by the farmer or the veterinarian for an etiologic diagnosis of respiratory signs. Samples were nasal and tracheobronchial swab specimens and lung tissues from pigs in Belgium and the Netherlands, collected during November 2019–December 2021. From those samples, we obtained 62 swine IAVs by virus isolation in MDCK cells. We determined their subtype by hemagglutination inhibition assays with reference antiserum that we obtained by double vaccination with inactivated virus vaccines (Appendix 1 Table) and a multiplex reverse transcription quantitative PCR, specific for the HA and NA (Table 1; Appendix 1 Table) (*4*). Two of those IAVs originated from pigs in the Netherlands; the other 60 were isolated from pigs in Belgium. Subtyping revealed 34 (54.8%) viruses of the H1N1 subtype, comprising 30 (48.4%) H1avN1av viruses, 3 (4.8%) pH1N1av viruses, and 1 (1.6%) H1huN1av virus. The second most dominant subtype was H1N2 with 27 (43.5%) viruses: 13 (21.0%) H1huN2 viruses, 13 (21.0%) pH1N2 viruses, and 1(1.6%) H1avN2 virus. We isolated 1 (1.6%) H3N2 virus.

**Table 1:** Overview of swine influenza A virus isolates from pigs in Belgium and the Netherlands, November 2019–December 2021

Subtype	Lineage	No. isolates/y			Total, N = 62
		2019, n = 4	2020, n = 34	2021, n = 24	
H1N1	H1avN1av	1	17	12	30
	pH1N1av	0	2	1	3
	H1huN1av	0	0	1	1
H1N2	H1huN2	2	8	3	13
	pH1N2	1	5	7	13
	H1avN2	0	1	0	1
H3N2	H3huN2	0	1	0	1

*av, European avian-like; hu, European human-like; p, 2009 pandemic

We selected 23 of the 62 swine IAV isolates for targeted whole-genome sequencing using nanopore sequencing (Table 2) (*5*–*7*). All sequences are available through GenBank (accession nos. OP445672–79, OP445741–812, OP458600–702). Sequencing of HA revealed that the isolates belonged to all 4 lineages reported in Europe: 10 H1av, 8 pH1, 4 H1hu, and 1 H3. For NA, all isolates of the H1N1 subtype had their NA derived from H1av viruses. Within the H1N2 subtype, 2 isolates derived their NA from N2s and 9 from N2g viruses. The 6 internal gene segments were derived from pH1 or H1av viruses or a combination of both. We designated the A/swine/Belgium/Gent-249/2020 genotype, previously reported by Chepkwony et al. (*7*), as AQ; we identified a genotype of isolate A/swine/Belgium/Gent-234/2020 and designated it as AR. The naming of both isolates is in accordance with the nomenclature system introduced by Watson (*8*) and updated by Henritzi (*9*). 

**Table 2:** Swine influenza A virus genotypes of 23 viruses isolated from pigs in Belgium and the Netherlands, November 2019–December 2021*

Subtype	Genotype	Surface genes			Internal genes						Total	
		HA	NA		PB2	PB1	PA	NP	M	NS		
H1N1	M	H1av	N1av		av	av	av	av	p	av	4	
	A	H1av	N1av		av	av	av	av	av	av	3	
	AB	H1av	N1av		av	av	av	p	av	av	1	
	AC	H1av	N1av		av	av	av	av	p	p	1	
	S	pH1	N1av		p	p	p	p	p	p	2	
	H	H1hu	N1av		av	av	av	av	av	av	1	
H1N2	AR	H1av	N2g		av	av	av	av	p	av	1	
	R	pH1	N2g		p	p	p	p	p	p	6	
	C	H1hu	N2s		av	av	av	av	av	av	1	
	AI	H1hu	N2g		av	av	av	av	p	av	1	
	AQ	H1hu	N2s		av	av	av	av	p	av	1	
H3N2	N	H3g	N2g		av	av	av	av	p	av	1	

*Nomenclature as described by Watson et al. (*7*). av, European avian-like; g, A/swine/Gent/1/1984-like; HA, hemagglutinin; hu, European human-like; M, matrix; NA, neuraminidase; NP, nucleoprotein; NS, nonstructural protein; p, 2009 pandemic; PA, polymerase; PB, polymerase basic; s, A/swine/Scotland/410440/1994-like.

**Figure 1:**
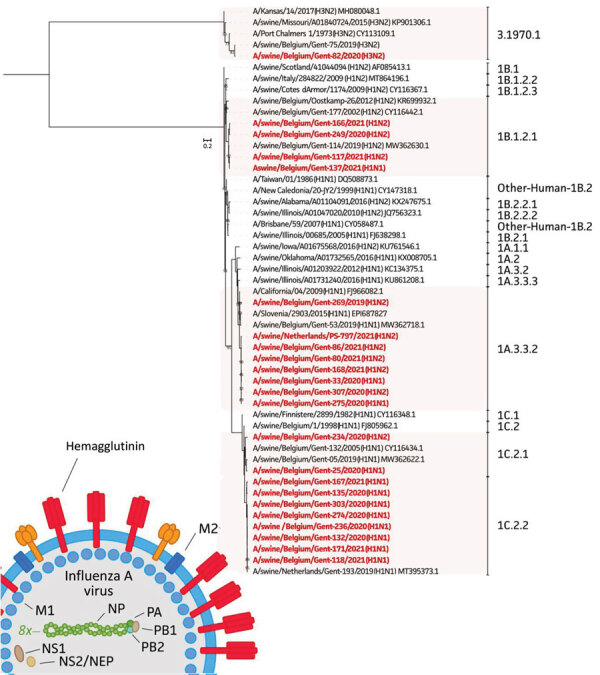
Phylogenetic tree based on the hemagglutinin nucleotide sequences of 23 swine influenza A isolates from pigs in Belgium and the Netherlands, November 2019–December 2021, and 28 swine and human influenza A reference viruses. We generated maximum-likelihood trees using IQ-TREE (http://www.iqtree.org) with the general time reversible plus invariable site plus FreeRate model and 1,000 ultrafast bootstraps. Sequences retrieved from GenBank or GISAID (https://www.gisaid.org) are identified by virus names and accession numbers. Bold text indicates study isolates. The clades of hemagglutinin are indicated; 8x means that 1 of the 8 total segments of the viral genome is shown. Scale bar represents the number of nucleotide substitutions per site per year. M, matrix protein; NEP, nuclear-encoded plastid RNA polymerase; NP, nucleoprotein; NS, nonstructural protein; PA, polymerase acidic; PB, polymerase basic.

**Figure 2:**
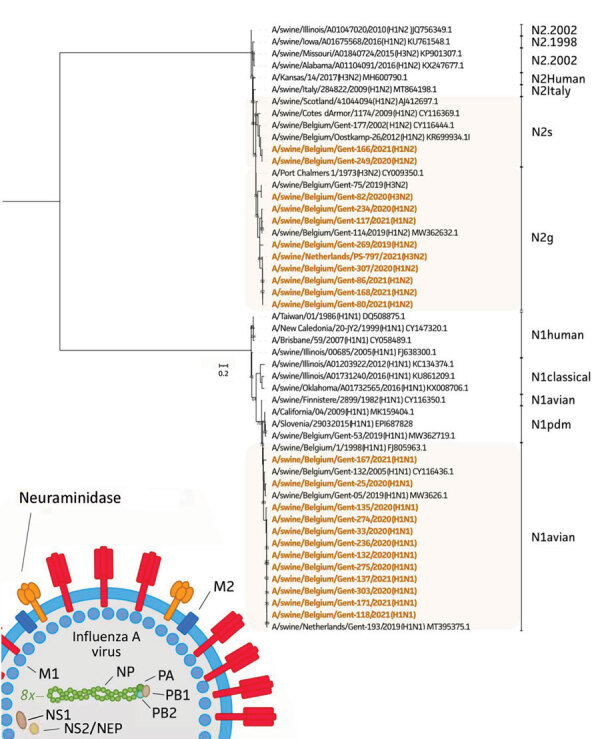
Phylogenetic tree based on neuraminidase nucleotide sequences of 23 swine influenza A isolates from pigs in Belgium and the Netherlands, November 2019–December 2021, and 28 swine and human influenza A reference viruses. We generated maximum-likelihood trees using IQ-TREE (http://www.iqtree.org) with the general time reversible plus invariable site plus FreeRate model and 1,000 ultrafast bootstraps. Sequences retrieved from GenBank or GISAID (https://www.gisaid.org) are identified by virus names and accession numbers. Bold text indicates study isolates. The lineage of neuraminidase is indicated; 8x means that 1 of the 8 total segments of the viral genome is shown. Scale bar represents the number of nucleotide substitutions per site per year. M, matrix protein; NEP, nuclear-encoded plastid RNA polymerase; NP, nucleoprotein; NS, nonstructural protein; PA, polymerase acidic; PB, polymerase basic.

For both HA and NA sequences, we performed maximum-likelihood phylogenetic analyses (*10*) (Figures 1, 2). We selected a total of 28 swine and human IAVs as reference viruses, including both historical and contemporary IAVs circulating in pigs as well as humans in Europe and North America; sources isolates were US Department of Agriculture, Worldwide Influenza Centre at Francis Crick Institute, US Centers for Disease Control, the Ploufragan-Plouzané-Niort Laboratory of the French Agency for Food, Environmental and Occupational Health & Safety(, and the Istituto Zooprofilattico Sperimentale delle Venezie. This analysis revealed that 8 of 10 H1av isolates belonged to clade 1C.2.2 and 2 isolates to clade 1C.2.1. In contrast, all 4 H1hu isolates belonged to clade 1B.1.2.1 and all 8 pH1 isolates to clade 1A.3.3.2. The NA genes grouped per lineage and the shorter horizontal branches suggest a slower evolution than the HA gene.

Two tracheobronchial swab specimens from swine farms, originating from Belgium and the Netherlands, tested IDV-positive via metagenomics in 2021 (*3*). The pigs had demonstrated mild upper respiratory signs. Cattle were present on both farms. The samples were investigated by virus isolation on swine testicle cells. We isolated IDV from the sample from the Netherlands and performed next-generation whole-genome sequencing. We designated the isolate as D/swine/Netherlands/PS-497/2021 (GenBank accession nos. OP474071–77); phylogenetic analyses of the HEF nucleotide sequence revealed that it belonged to the D/660 lineage and clustered together with recent bovine IDV isolates from Italy (Appendix 2). In addition, a BLAST homology search (http://www.fludb.org) of all gene segments showed no evidence of reassortment and confirmed the close relationship to D/660.

## Conclusions

This study is a follow-up of a previous surveillance study in Belgium and the Netherlands during 2014–2019 (*7*). The H1av lineage was predominant in both studies and accounted for roughly half of all swine IAV isolates. A major change, however, was the increase in clade 1C.2.2 isolates from 23.8% in 2014–2019 to 80.0% in 2019–2021 (*7*). Of note, recent serologic investigations of human antibodies against H1 swine IAVs pointed toward a relatively greater zoonotic risk for H1av viruses compared with European H1hu or pH1 viruses (*11*).

The second most predominant lineage was the pH1 lineage (25.8%). Until 2018, this lineage was widespread in the United Kingdom and Poland, whereas prevalence in other countries in Europe was ≈5% (*8*,*9*). Most pH1 viruses in this study were reassortants in which the pN1 was replaced by N2, a trend described previously (*8*). The increased frequency of pH1 swine IAVs explains the emergence of second-generation reassortants between this lineage and the long-existing H1av lineage in swine in Europe as well as in Asia. Some specific reassortant genotypes with H1av surface proteins and pH1 internal genes that have been found in Asia were announced as a pandemic threat (*12*). However, it remains unclear whether they are higher on the pandemic risk scale than other H1av reassortants because of the lack of comparative data. The increase in pH1N2 swine IAVs might also play a role in the low number of H3N2 swine IAVs in Belgium and the Netherlands; this connection was previously described as a possible result of immunity against N2 influencing the prevalence of both virus lineages and favoring the lineage with the greatest genetic diversity in swine (*8*).

In summary, we report IDV isolation in swine in the Netherlands and circulation of lineage D/660 in swine in Europe that is genetically related to bovine IDV. Trombetta et al. previously suggested circulation of the D/660 lineage in swine in Italy, based on the detection of antibodies in swine veterinarians in 2004 (*13*). The finding of bovine-related IDV strains in those pigs is unsurprising because cattle were present on both farms, suggesting a potential interspecies transmission. Although the phylogenetic analysis seems to confirm that the IDV HEF evolutionary rate is slower than that of IAV HA and NA (*2*) (Appendix 2), we note that IDV emerging virus in swine may have zoonotic potential (*13*,*14*). Therefore, this study supports the hypothesis that pigs and swine influenza viruses should be a high priority for surveillance for pandemic threats (*15*).

Appendix 1 Reference sequences used in study of influenza A and D viruses in swine in Belgium and the Netherlands, 2019–2021.

Appendix 2 Additional information about influenza A and D viruses in swine in Belgium and the Netherlands, 2019–2021.
