# Supplementary material for: Surveillance and Genomic Characterization of Influenza A and D Viruses in Swine, Belgium and the Netherlands, 2019–2021
Source: Emerg Infect Dis. 2023 Jul;29(7):1459–64. doi: 10.3201/eid2907.221499 (PMC10310360; doi:10.3201/eid2907.221499)
Supplement: Appendix 2 — Additional information about influenza A and D viruses in swine in Belgium and the Netherlands, 2019–2021. [file 22-1499-Techapp-s2.pdf]

Article DOI: http://doi.org/10.3201/eid2907.221499

Surveillance and Genomic Characterization of Influenza A and D Viruses in Swine, Belgium and the Netherlands, 2019–2021

Appendix 2

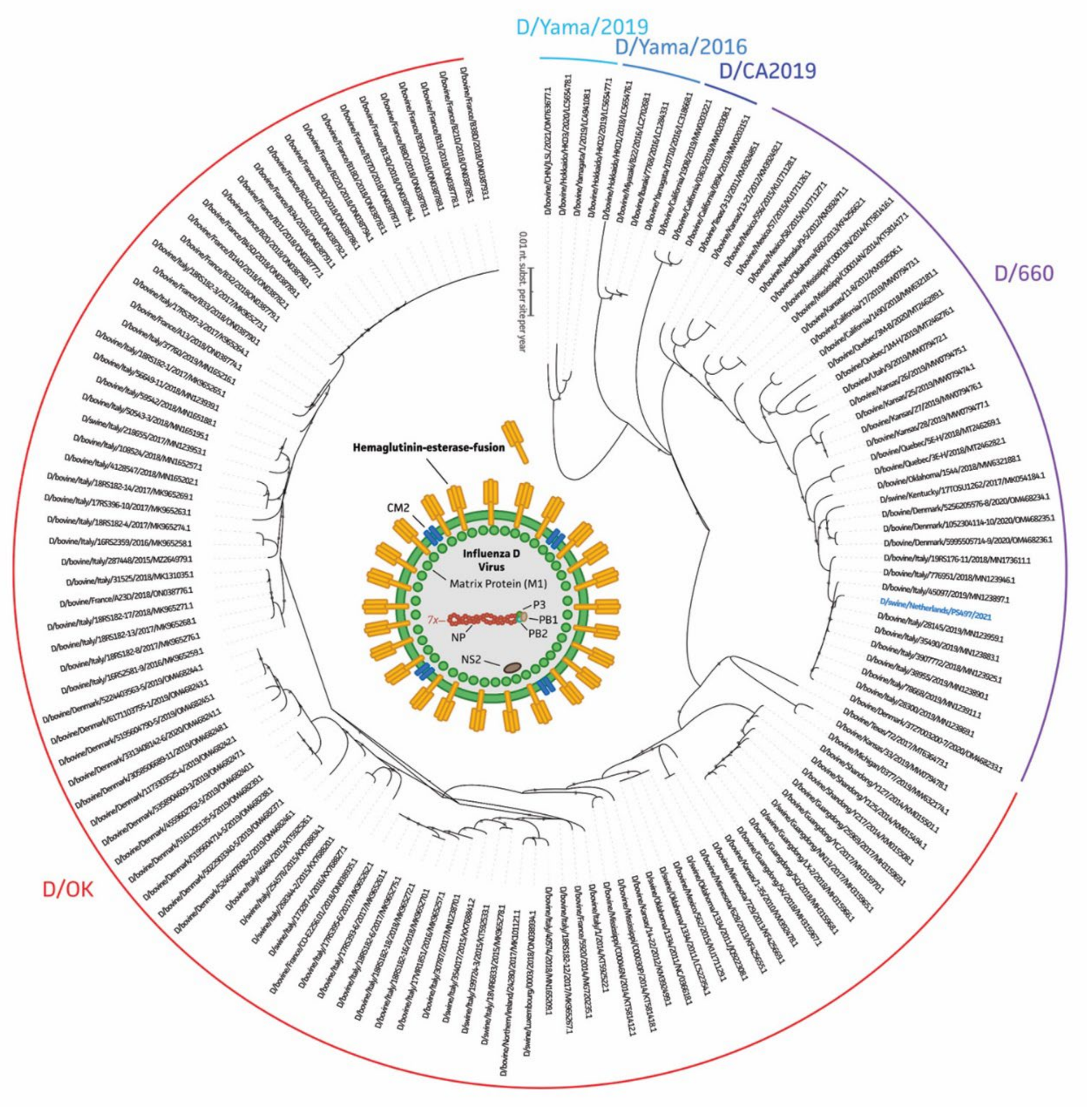

Appendix 2 Figure. Phylogenetic tree based on the hemagglutinin-esterase fusion protein nucleotide sequences of influenza isolate D/swine/Netherlands/PS-497/2020 (blue) and the 142 swine and bovine IDV HEF nucleotide sequences available in GenBank. identified by their virus name and accession

number. We generated maximum-likelihood trees using IQ-TREE (http://www.iqtree.org) with the general time-reversible plus invariable site plus FreeRate model and 1,000 ultrafast bootstraps. The influenza D lineage is indicated. Scale bar represents the number of nucleotide substitutions per site per year. M, matrix protein; NP, nucleoprotein; NS, nonstructural protein; PB, polymerase basic; P3, polymerase 3.
